# Milk in Febrile Diseases

**Published:** 1874-10

**Authors:** B. H. Washington


Milk in Febrile Diseases.—*By B. H. Washington, M.D.*— * * * The class of cases in which milk may prove beneficial, and that class in which its use will prove decidedly injurious, can be very readily defined, and I do not think a spare hour could be better spent than in calling the attention of young practitioners to the subject.

Prof. Campbell says: "As in the nervous system we recognize two grand departments, viz: 1st. The cerebro-spinal system, all the normal actions of which are subject to cessations and interruptions; and 2d. The ganglionic system, all the normal actions of which are of a continuous and uninterrupted character; so in the manifestations of febrile diseases, do we distinctly recognize the two grand distinguishing characteristics respectively typifying the normal actions of these two systems of nerves; thus a character of *paroxysms* obtains in certain cases, while a character of *continuousness* as plainly marks the other." * * * "We would therefore announce, as our classification of febrile diseases, two grand divisions of fevers, corresponding with the two grand divisions of the nervous system, thus:

1. "Cerebro-spinal fevers—all *paroxysmal.* The secretions and nutrition only *secondarily* affected.

"2. Ganglionic fevers—all *continued.* The secretions and nutrition *primarily* affected.

"1. Under the head of *Cerebro-Spinal Fevers* we would place the whole family of paroxysmal fevers, whatever type they may assume, and also the various forms of neuralgia which are nearly always intermittent, as well as *sthenic* forms of traumatic fever, together with the fever accompanying simple pharyngitis, pneumonitis, dysentery and many other diseases of malarial districts.

"2. Under the head of *Ganglionic Fevers*, or *Fevers of the Secretory System of Nerves*, we think we find ample ground for bringing together many diseases heretofore widely estranged from each other. Thus, as the archetypal forms of ganglionic fevers, we place at the head of the list typhus and typhoid fevers; then, allied to these, in various degrees of affinity, but all *equally* in the one essential element that they present themselves as manifestations of disease through the ganglionic system, are variola, scarlatina, rubiola, varicella, and many other forms of eruptive fevers heretofore not classified by nosologists.

"All of these last diseases are marked by fever of a *continued* or non-paroxysmal character; all present marked aberrations of nutrition and secretion, and each has its own peculiar eruptive character; and furthermore, each one is definitely self-limited in its duration, no remedial interference having as yet been found competent to arrest or shorten their progress."

Now, my experience has been that, in the ganglionic fevers, the use of milk is highly beneficial, and, in the cerebro-spinal fevers, the use of milk is decidedly injurious.—*Nashville Journal of Medicine and Surgery.*

